# A Hemodynamic Analysis of the Thrombosis Within Occluded Coronary Arterial Fistulas With Terminal Aneurysms Using a Blood Stasis Model

**DOI:** 10.3389/fphys.2022.906502

**Published:** 2022-05-23

**Authors:** Xudong Jiang, Haoyao Cao, Zijian Zhang, Tinghui Zheng, Xiaoqiang Li, Peng Wu

**Affiliations:** ^1^ Artificial Organ Technology Laboratory, School of Mechanical and Electric Engineering, Soochow University, Suzhou, China; ^2^ Department of Vascular Surgery, Nanjing Drum Tower Hospital Clinical College of Traditional Chinese and Western Medicine, Nanjing University of Chinese Medicine, Nanjing, China; ^3^ College of Architecture and Environmental Engineering, Sichuan University, Chengdu, China; ^4^ Sichuan University Yibin Park/Yibin Institute of Industrial Technology, Yibin, China

**Keywords:** coronary arterial fistulas, thrombosis, blood stasis, occlusion position, CFD

## Abstract

**Objective:** The aim of this study is to numerically evaluate thrombosis risk within occluded coronary arterial fistulas (CAF) with terminal aneurysms, and provide guidance in choosing occlusion positions, with clinical observations as reference.

**Method:** Four patients with CAF were studied, with different occlusion positions in actual treatments. Hemodynamics simulations were conducted, with blood residue predicted using the blood stasis model. Three types of models (untreated model, aneurysm-reserved model and aneurysm-removed model) were studeid for each patient. Four metrics, i.e., proportion of high oscillatory shear index (OSI), area of high OSI, old blood volume fraction (OBVF)) and old blood volume (OBV) was obtained to distinguish the thrombosis risk of different treatments (proximal or distal occlusion), comparing with the follow-up CTA.

**Results:** For all the postopertive models, the high OBVF, high OSI(>0.3) and low time-averaged wall shear stress (TAWSS) regions were mainly at the distal fistula, indicating these regions were prone to thrombosis. The regions where blood residue remains are roughly regions of high OSI, corresponding well with clinical observations. In contrast, TAWSS failed to distinguish the difference in thrombosis risk. Absolute values (area of high OSI, OBV) can better reflect the degree of thrombosis risk between treatment types compared with percentage values (proportion of high OSI, OBVF). By comparing with the actual clinical treatments and observations, the OBV is superior to the area of high OSI in determining treatment type.

**Conclusion:** The OBV, a volumetric parameter for blood stasis, can better account for the CAF thrombosis and reflect the degree of blood stasis compared with OSI or TAWSS, is a more appropriate metric for thrombosis in the fistula. Together with morphological parameters, the OBV could guide clinicians to formulate more appropriate surgical plans, which is of great significance for the preoperative evaluation and treatment prognosis of CAF patients.

## Introduction

Coronary arterial fistula (CAF) is defined as an abnormal connection between one of the coronary arteries and a heart chamber or another blood vessel, such as the coronary vein, pulmonary artery, superior vena cava, or bronchial artery etc. (Qureshi, 2006; Iskandrian et al., 1978; Verdini et al., 2016; Amin et al., 2001). CAF is relatively rare, with a prevalence of 0.002% in the general population. Even among patients undergoing coronary angiography or coronary computed tomography angiography, the prevalence of CAFs is only approximately 0.05–0.9% (Karazisi et al., 2017). Most patients have no symptoms when they are young. However, with age, clinical symptoms gradually occur, such as exertional dyspnea, chest pain, infective arteritis, etc. (Firouzi et al., 2021). If combined with coronary arteries dilation or aneurysms, it may cause serious complications such as acute myocardial infarction, cardiac failure, rupture, arrhythmias, etc (Qureshi, 2006). Therefore, immediate clinical intervention is necessary.

The treatments of CAF mainly include surgical ligation, transcatheter closure (TCC) and drug therapy (Kilic et al., 2008; Akcay et al., 2009; Jama et al., 2011; Centella et al., 2016; Haweleh et al., 2018; Firouzi et al., 2021). A recent study proposed that in patients with medium‐ to large-sized fistulas, irrespective of symptoms, closure (either surgical ligation or TCC) is recommended (Firouzi et al., 2021). The common method is to occlude the terminal of the fistula, while retaining small branches to maintain the blood supply of the myocardium (Kilic et al., 2008; Jama et al., 2011) and improve the phenomenon of stealing blood. However, postoperative patients, especially those with coronary arteries dilation and aneurysms, are prone to thrombus formation in the occluded fistula, which may induce serious consequences such as angina, myocardial infarction, etc. (Saboo et al., 2014). Therefore, for CAF patients undergoing intervention, warfarin is recommended postoperatively. Furthermore, if thrombosis is observed in the occluded fistula, long-term anticoagulation with warfarin is necessary (Karazisi et al., 2017). Unfortunately, long-term anticoagulation therapy is often accompanied by the risk of bleeding. If the patient is young, it will directly affect the patient’s life safety and long-term quality of life (Pu et al., 2016). Therefore, surgical procedures need to be optimized to reduce the risk of thrombosis. Some clinicians have suggested that in CAF patients with aneurysms, the use of proximal aneurysm closure is effective in reducing the incidence of postoperative fistula thrombosis (Reul et al., 2002; Gowda et al., 2011). However, these are only clinical retrospective studies, more systematic quantitative studies will be needed.

Hemodynamics play an important role in the initiation, formation, and aggregation of thrombosis (Jama et al., 2011; Skorczewski et al., 2013; Liang et al., 2015). In recent years, computational fluid dynamics (CFD) studies have been employed to study hemodynamics (Wu et al., 2020, 2021, 2022; Huo et al., 2021) and evaluate risk of thrombosis in abdominal aortic aneurysm, atrial appendages, aortic dissection, etc. (Georgakarakos et al., 2009; Menichini et al., 2017; Bosi et al., 2018; García-Isla., 2018). In our previous studies, four patient-specific CAF models were studied using CFD (Cao et al., 2019,^2020^), with conventional hemodynamics parameters such as time-averaged wall shear stress (TAWSS) and oscillatory shear index (OSI) to evaluate the thrombosis risk. The results showed that a proximal occlusion to remove the terminal aneurysm may potentially reduce the post-operative thrombotic risk (Cao et al., 2019), while a terminal occlusion of CAF fistula may increase the risk of thrombosis.

In this study, a two-fluid blood stasis model (Jiang et al., 2021a; Jiang et al., 2021b; Dai et al., 2021), which can locate the regions of blood stasis both in space and time was used together with conventional hemodynamic parameters such as OSI and TAWSS as well as morphological parameters, to access the influence of occlusion positions to the risk of thrombosis in the fistula of CAF patients. The approach employed in this study can better assist clinicians choosing the correct surgical plan, so that myocardial blood supply can be improved while minimizing the risk of thrombosis in the fistula.

## Materials and Methods

### Subject Data

**FIGURE 1:**
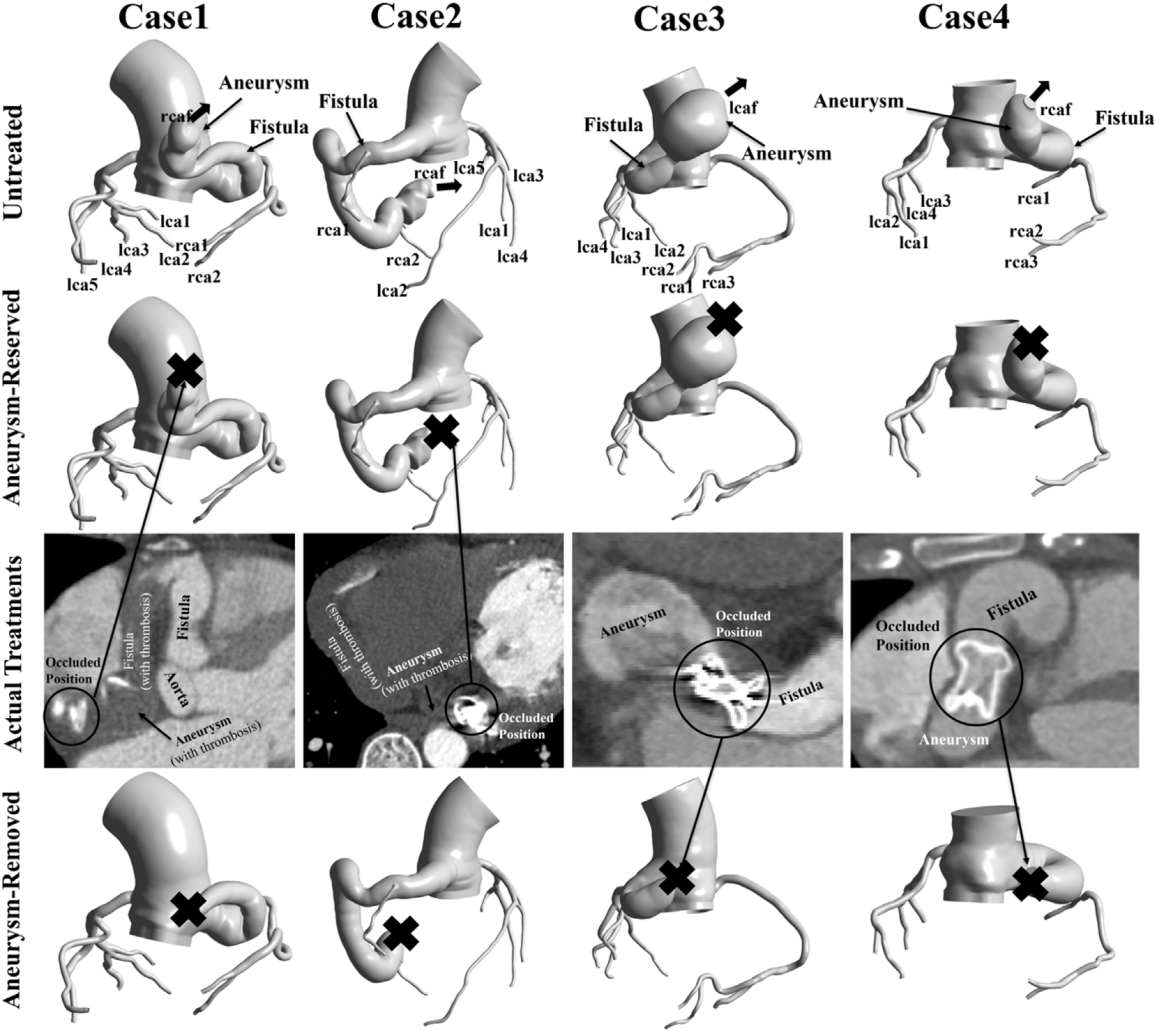
Patient-specific models reconstructed from CTA data. “Aneurysm-reserved model” and “Aneurysm-removed model” referred to the proximal or distal occlusions. In actual treatments, case1 and case2 had distal occlusions, while case3 and case4 had proximal occlusions. “X” indicates the occluded position of the model.

This study used the same patient-specific models as our previous research (Cao et al., 2020). Four patient-specific models were reconstructed based on computed tomography angiography (CTA) images, as shown in Figure 1. These four patients were treated between May 2015 and March 2018, with three right coronary fistulas and one left coronary fistula. The parameters of the models are shown in Table 1. Patient approval and informed consent were waived off since it is an observational and retrospective study with anonymized data.

**TABLE 1:** Patient specifics.

	Gender	Age	Origin and Drainage	D_E_	L_F_	D_MA_	L_A_	V_F_	V_A_
Case 1	Female	70	RCA - RA	16.3	130.2	40.8	39	20,262	7,055
Case 2	Male	25	RCA - LV	17.6	227.6	20.5	31.1	33,230	923
Case 3	Female	46	LCA - RA	14.2	105.5	45.7	42.2	41,788	35,367
Case 4	Female	27	RCA - RA	21.5	99.8	23.5	31.2	32,343	9,810

RCA, right coronary artery; LCA, left coronary artery; RA, right atrium; LV, left ventricle. D_E_, average diameter of fistula entrance (mm); L_F_, length of fistula (mm); D_MA_, max diameter of aneurysm (mm); L_A_, length of aneurysm (mm); V_F_, volume of fistula (mm^3^); V_A_, volume of aneurysm (mm^3^).

In the actual treatment, Patient one and Patient two had distal occlusions, while Patient three and Patient four had proximal occlusions. In this study, each patient underwent virtual occlusion of the distal and proximal aneurysm. Therefore, three types of models, i.e., untreated model, aneurysm-reserved model and aneurysm-removed model (as shown in Figure 1) were studied for each patient, to simulate three treatments, namely, no treatment, proximal and distal occlusions.

### Mesh

**FIGURE 2:**
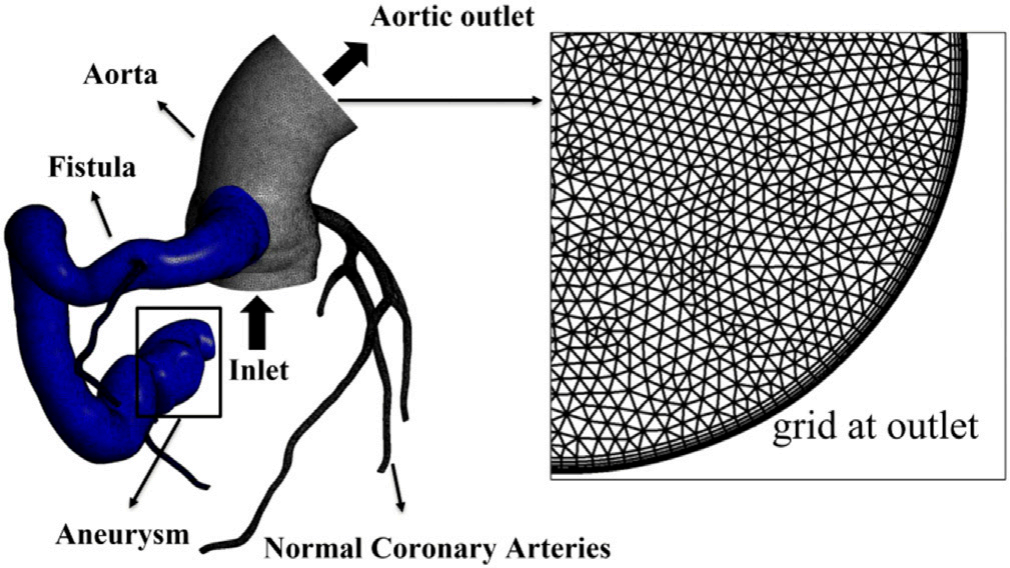
Computational domain and mesh of case 2, with local grid at the aortic outlet showing five boundary layers. The fistula region is indicated by blue part.

The computational domains of all the four models were divided into two separate parts: the fistula regions and the rest, to facilitate the quantitative analysis of blood stasis in the fistula. Structured grids of 1–1.5 million elements were generated by using commercial software Ansys Meshing (Ansys, Inc., Canonsburg, PA, United States). Five grid layers were added to all the arterial walls to properly resolve the boundary layer, as shown in Figure 2.

### Boundary Conditions

**FIGURE 3:**
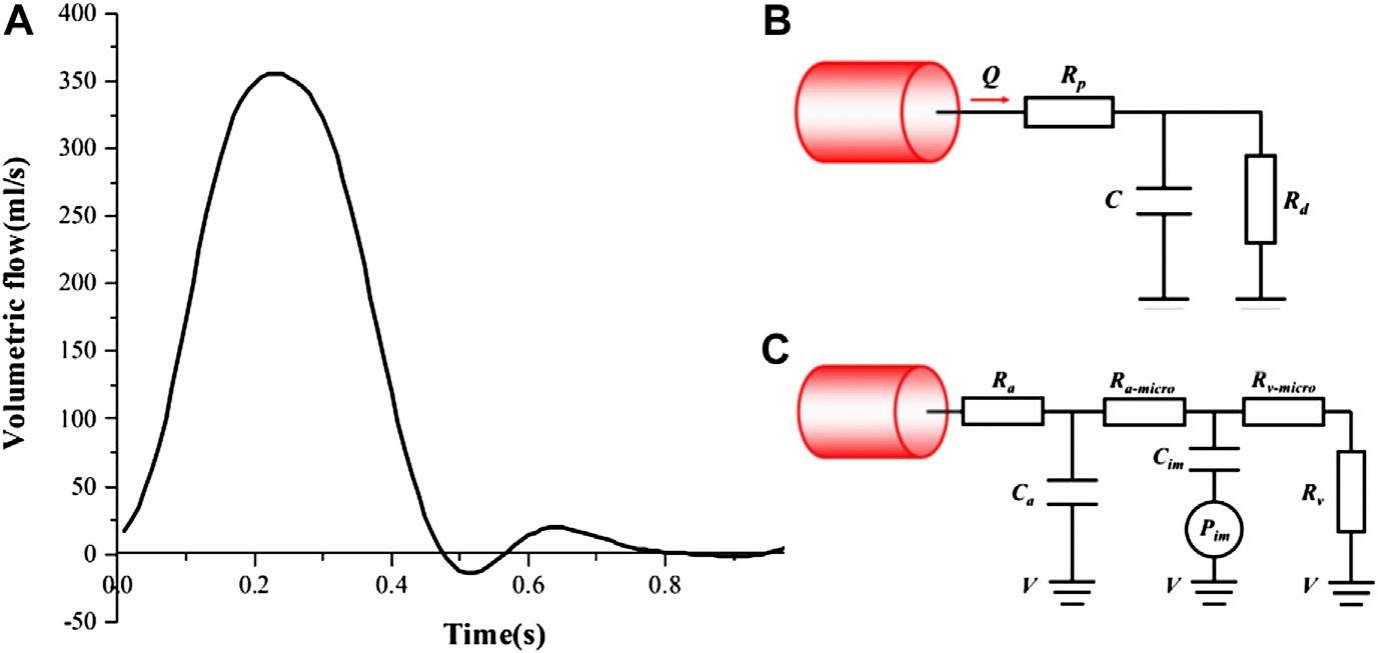
Boundary conditions: **(A)** The inlet velocity profile; **(B)** Windkessel RCR boundary conditions; **(C)** Lumped parameter boundary conditions.

As shown in Figure 3A, a time-varying volumetric flow rate extracted from the literature was applied at the inlet of each model with a period of 1s (Cheng et al., 2014). Windkessel RCR boundary conditions (Figure 3B) and lumped parameter network (LPN) coronary model (Figure 3C) were applied at the aortic outlet and the coronary outlets, respectively (Reul et al., 2002; Cheng et al., 2014; Cao et al., 2020). The total resistance (R_total_) of each case were shown in Table 2, and in the Windkessel model, the distal resistance was set to 0.91R_total_, the proximal resistance was set to 0.09 R_total_, and the capacitance was set to 0.001 cm^5^/dyne. The outlet of the fistula was set to “wall” to represent the occlusion. All the walls were assumed to be rigid with no slip conditions.

**TABLE 2:** Total resistance.

Outlets/Total Resistance (Pa*S/m³)	Case 1	Case 2	Case 3	Case 4
aorta	2.3171*10^8^	2.7095*10^8^	1.9165*10^8^	2.8273*10^8^
rcaf	6.8721*10^8^	3.7588*10^8^	—	3.9499*10^8^
rca1	6.2281*10^9^	2.7159*10^10^	4.3397*10^10^	1.2955*10^10^
rca2	1.2908*10^10^	2.7642*10^10^	2.4138*10^10^	9.8035*10^9^
rca3	—	2.4753*10^10^	1.1231*10^10^	1.2419*10^10^
lcaf	—	—	3.7154*10^8^	—
lca1	7.7446*10^9^	2.6813*10^10^	3.2448*10^10^	9.3985*10^9^
lca2	7.3605*10^9^	1.9396*10^10^	2.2431*10^10^	1.5694*10^10^
lca3	3.9235*10^9^	1.8043*10^10^	1.9728*10^10^	1.7522*10^10^
lca4	1.4137*10^10^	1.9588*10^10^	2.7451*10^10^	6.9969*10^9^
lca5	1.0121*10^10^	2.6045*10^10^	—	—

### Numerical Simulations

In this study, all the simulations were transient and conducted using the commercial software Ansys Fluent (Ansys, Inc, Canonsburg, PA, United States). Blood was regarded as incompressible Newtonian fluids, with density of 1055 kg/m^3^, and dynamic viscosity of 3.5 × 10^−3^ Pa s. Since the flow in aorta might lie in turbulence flow regime (Bigras 2020; Mandell et al., 2021; Manchester et al., 2022), the RNG k-ε model was employed to solve for turbulence. The near-wall treatment was set as standard wall function. A second-order implicit backward Euler scheme was chosen for temporal discretization, with a fixed time-step of 10 ms so that per cardiac cycle was resolved using 100 time steps. Maximum 50 sub-iterations were used for each physical time step, and the maximum RMS residual was set to 10^−5^ as a convergence criterion. First, unsteady single-fluid simulations were carried out for about 10 cardiac cycles to get statistically converged flow field.

Then, a two-fluid model for blood stasis was employed to simulate the process of blood washout and stasis (Jiang et al., 2021a; Jiang et al., 2021b; Dai et al., 2021). Simulation of blood stasis continued from the converged single-fluid flow field. A new fluid was defined with the same material properties as the existing blood in the computational domain. Then, the new fluid and the existing blood were defined as two fluids: the “new” and “old” blood. All the computational setup was the same as the single-fluid runs, except that the VOF method was employed to solve for the two-fluid flow field. The volume fraction of the new blood was set as one at the inlet, while that of the old blood was 0 at the inlet. The surface tension was set to be 0. As time evolves, the new blood will gradually replace the old blood. The location of and volume fraction of old blood can be tracked and monitored over time. Convergence criteria was set that the old blood volume fractions (OBVFs, defined as the ratio of residual blood volume to fistula volume) dropped within 1% in the past 10 cardiac cycles for all cases. Due to the disparity in fistula volume and treatment for different cases, 10–60 s (cardiac cycles) were needed to reach convergence. All computations were carried out on a 192-core cluster equipped with 16 Intel Xeon E5-2680 v3 CPUs. The single-fluid simulations normally converged within 1 h, while the blood stasis simulations took less than 1 day.

## Results

### Flow Pattern

**FIGURE 4:**
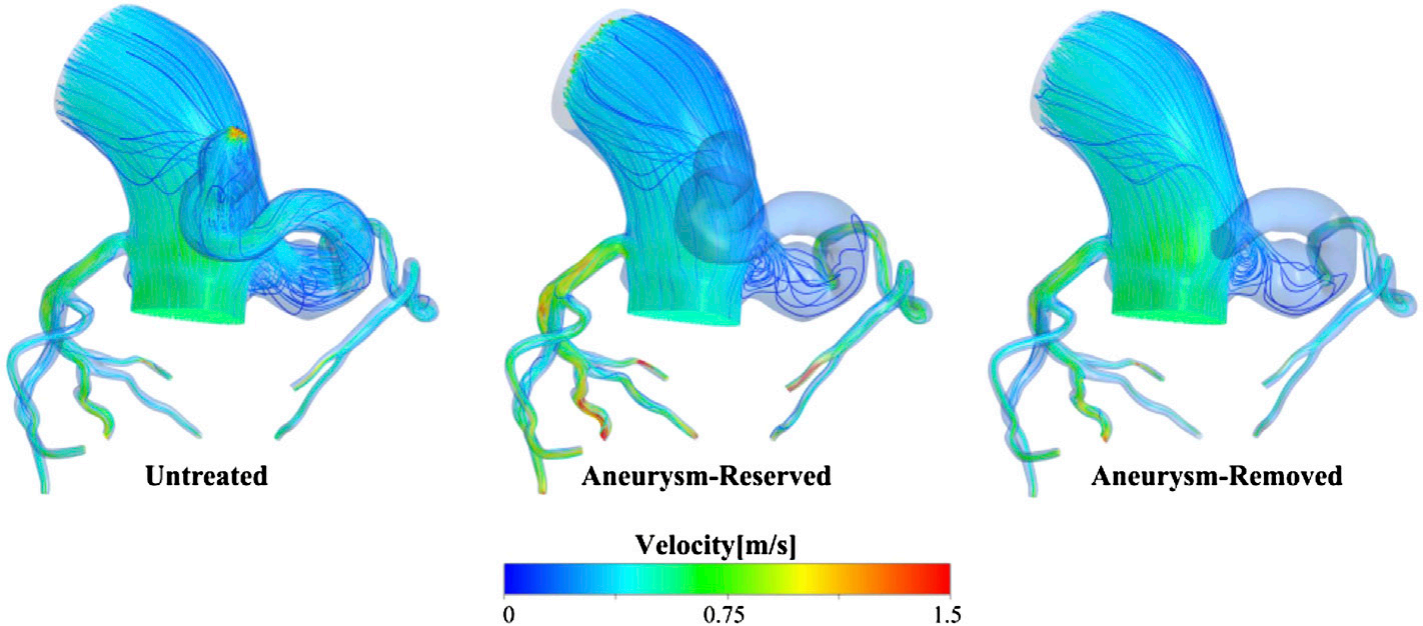
Streamlines of case 1 at systolic peak.

The ratio of flow rate at the aortic outlet to the inlet flow rate is shown in Table 3, which shows that proximal occlusion of aneurysm indeed effectively improved the phenomenon of blood stealing. The flow streamlines of case 1 at systolic peak were shown in Figure 4, after the proximal occlusion, the blood flow in the fistula was significantly less than that of the untreated models.

**TABLE 3:** Percentage (%) of blood flow rate at the aortic outlets.

Case/Model	Untreated	Aneurysm-Reserved	Aneurysm-Removed
Case 1	69.52	72.72	87.93
Case 2	60.83	91.90	97.22
Case 3	77.82	90.36	95.54
Case 4	57.64	89.16	97.28

### Results of TAWSS and OSI

**FIGURE 5:**
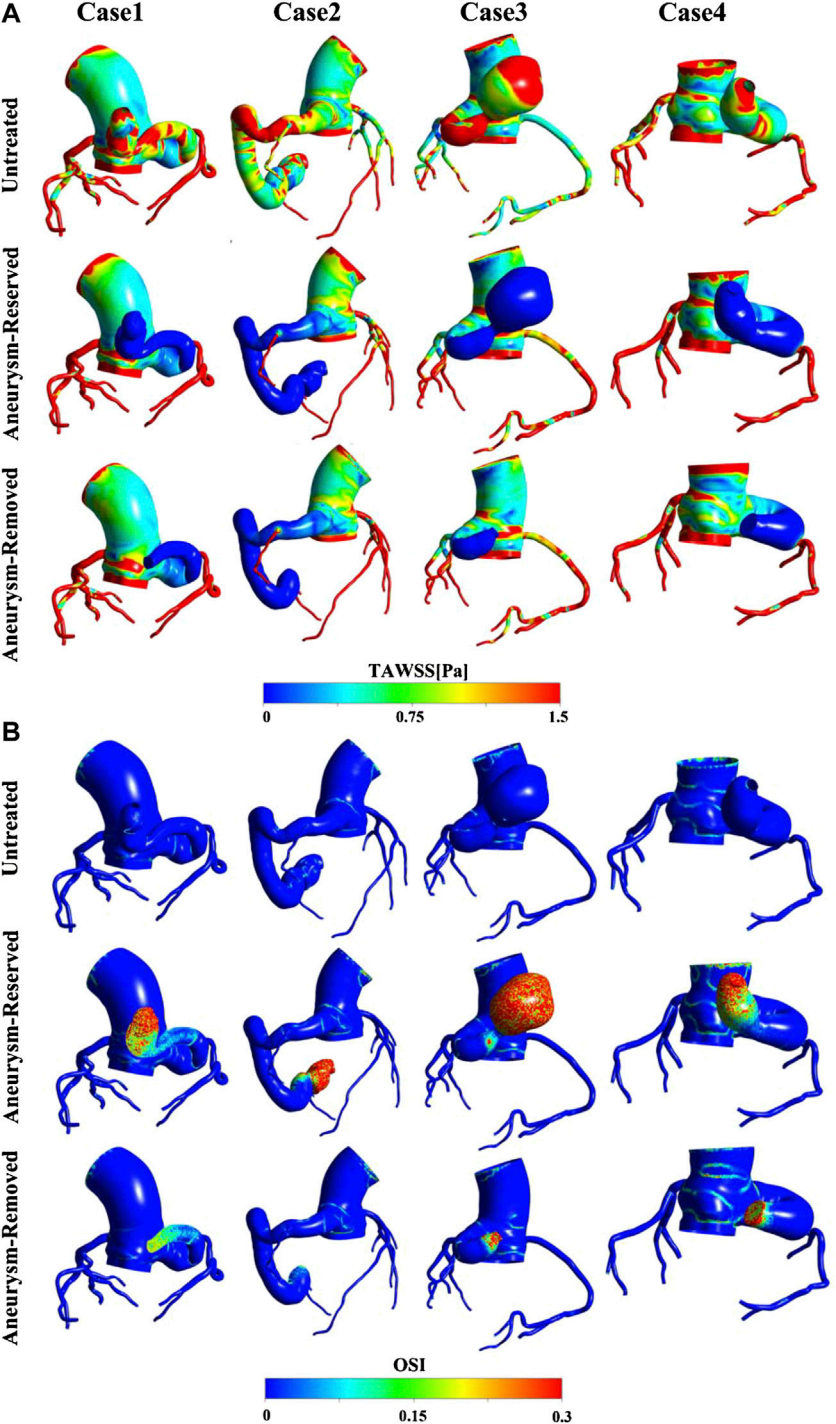
**(A)** TAWSS contours; **(B)** OSI contours.

TAWSS and OSI are hemodynamic parameters which are commonly used as metrics to evaluate thrombosis in arterial system. It is generally believed that high OSI (>0.3) and low TAWSS (<10 dyne/cm^2^) are associated with thrombosis (Nerem et al., 1998; Ward et al., 2001; Himburg et al., 2004; Katritsis et al., 2012). Figure 5A shows that TAWSS was generally low in the distal fistula of all the post-treated models, so the results of TAWSS proved that the occlusion treatment will be more prone to thrombosis. However, it can be observed that there was almost no difference in the level of TAWSS in the fistula between different patients and treatments. Thus, TAWSS is not a proper metric for the risk of thrombosis in this scenario. Figure 5B shows the OSI contours. In contrast to TAWSS, the OSI was high in the distal fistula of the post-treated models, and low near the junction with aorta. It is also worth noting that the area of high OSI varies greatly among cases, and regions of high OSI can always be observed near the occlusion positions.

### Results of Blood Stasis

**FIGURE 6:**
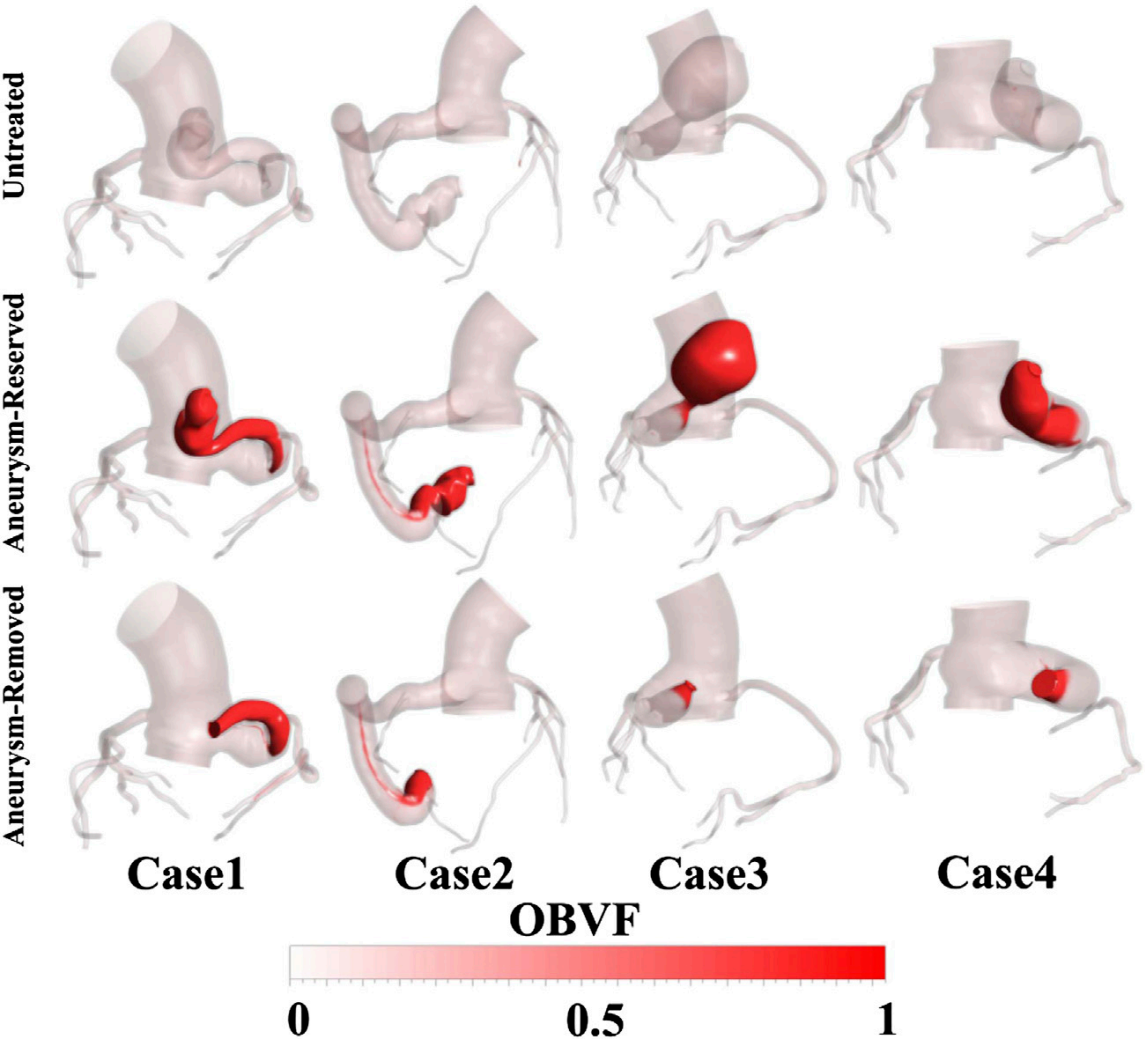
The OBVFs of all models, with the red color representing the blood residue.

The OBVFs of all models is shown in Figure 6, where the red color represents the blood residue (old blood), and the transparent color indicates where the old blood had been replaced by new blood. In all models, the old blood in the aorta was eventually replaced by new blood. Comparing the distribution of blood residue, there was almost no blood residue in the fistula of the untreated model, while for the post-treated model, the blood residue at the distal fistula was significantly higher than other regions, indicating that this region was prone to blood stasis. This phenomenon was more obvious in the aneurysm-reserved models and it can be observed that the distal occlusion was associated with the most serious blood stasis, among the three ways fistulas were treated. The distributions of blood residue in the aneurysm-removed and aneurysm-reserved models were generally consistent, except for the aneurysm. As shown in Figure 6, for case3 and case4, the high OBVF regions (red part) in proximal occluded models were significantly smaller than that in distal occluded models. However, for case1 and case2, the differences between two occluded positions were not obvious.

### Comparison With Clinical Follow-Ups

**FIGURE 7:**
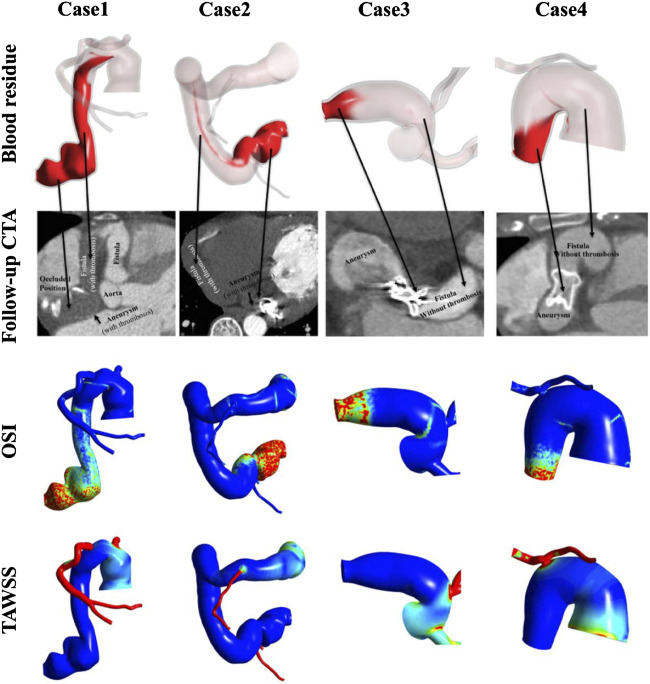
Comparison of blood residue, the follow-up CT after the occlusion operation, and contours of OSI and TAWSS (in order from top to bottom) of the models under actual treatments.

Figure 7 shows the clinical follow-ups after a short period of time, together with the predicted old blood residue, OSI and TAWSS contours of the actual treatment models. It can be observed that severe thrombosis occurred in the fistulas of case1 (1 week) and case2 (9 months) after the distal aneurysm occlusions, while no thrombosis was found in the fistulas of case3 (3 weeks) and case4 (1 month) after the proximal aneurysm occlusions. It can be observed that the location of thrombosis was consistent with the predicted old blood residue. Moreover, the regions where blood residue remained were also roughly regions of high OSI (>0.3). In contrast, the TAWSS contours failed to reflect the actual thrombus in the fistula.

### Quantitative Comparison Between Patients and Treatments

**FIGURE 8:**
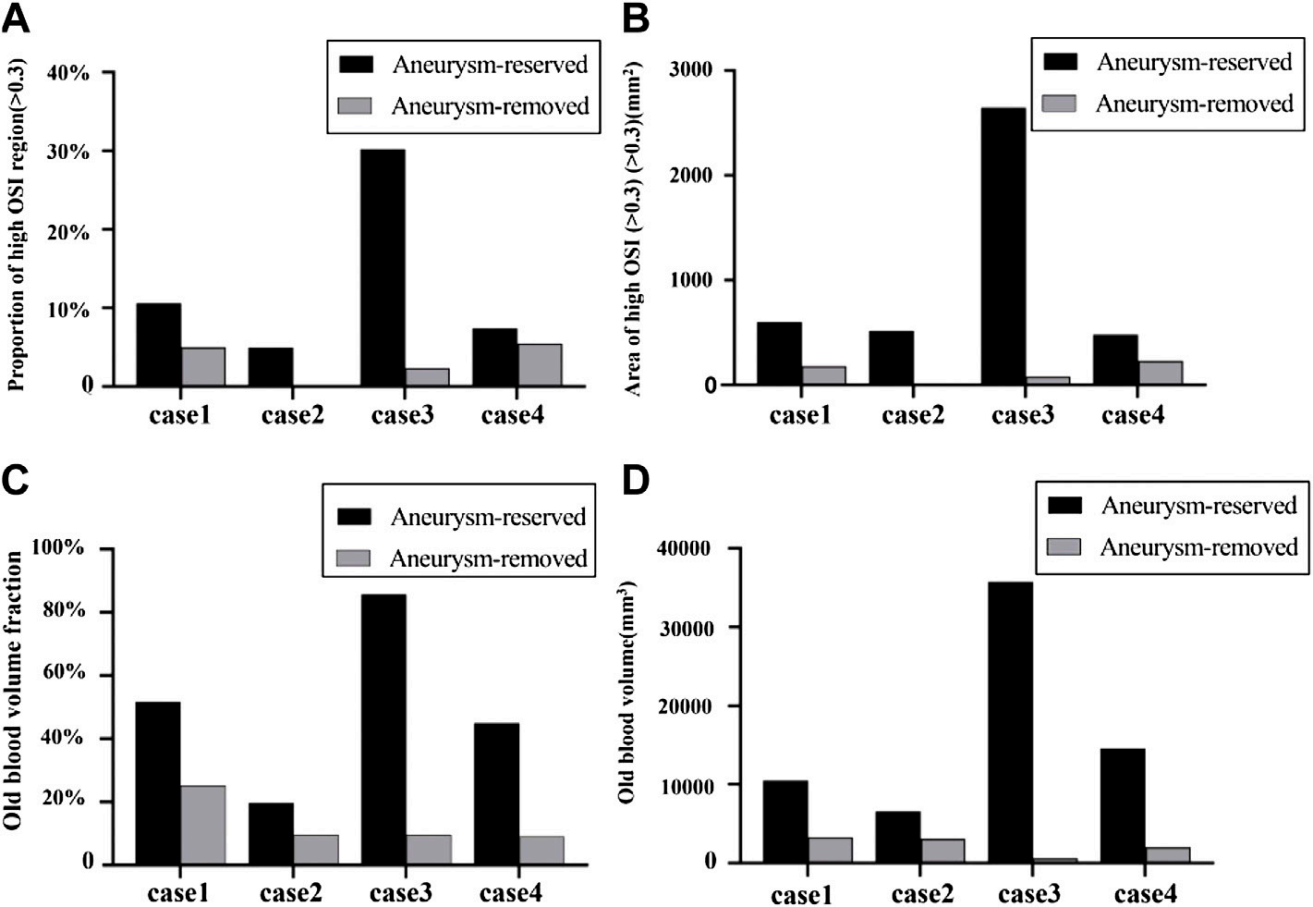
Quantitative comparison between patients and treatments in terms of OSI and blood stasis: **(A)** proportion of high OSI (>0.3) regions; **(B)** area of high OSI (>0.3) for aneurysm-reserved and aneurysm-removed models; **(C)** OBVF (old blood volume fraction, defined as the ratio of volume of blood residue and volume of the fistula); **(D)** OBVs (old blood volume).

As shown in Figure 8, all the four metrics (proportion of area of high OSI, area of high OSI, OBVF and OBV) distinguished the thrombosis risk of distal and proximal aneurysm occlusions well. All the four metrics decreased for aneurysm-removed models compared to aneurysm-reserved models. Thus, all of them could distinguish the thrombosis risk of distal and proximal aneurysm occlusions for each individual patient.

However, as aforementioned, severe thrombosis occurred in the fistula of case2 after the distal aneurysm occlusions and no thrombosis was found in the fistulas of case4 after the proximal aneurysm occlusions, while the proportions of area of high OSI were roughly the same (cf. Figure 8A). On the contrary, as shown in Figure 8B, the area of high OSI could distinguish the degree of thrombosis between these two scenarios, so could the metrics of blood stasis (OBVF&OBV).

The degree of difference in the thrombosis risk for the aneurysm-reserved and aneurysm-removed models also determines the type of treatment (proximal occlusion or distal occlusion) to be chosen. Figures 8C, D show that, case3 and case4 are more suitable for proximal aneurysm occlusion compared with case1 and case2, for which the OBVF and OBV between the two treatments is not much different. For case1, the OBV (3322 mm^3^) is the highest among all aneurysm-removed models, which means even under proximal occlusion, the thrombosis risk would still be high, and postoperative long-term anticoagulation is inevitable. Moreover, case1 has the second largest aneurysm size, proximal occlusion will greatly reduce the blood supply of the myocardium. Therefore, case1 is more suitable for distal aneurysm occlusion. For case2, the difference in OBV between the distal and proximal occlusions is less than 50%. Due to the largest fistula size and the smallest aneurysm size, removal of the aneurysm will not have a big impact on the blood supply. Therefore, either treatment can be applied to case2, subject to patient conditions and intraoperative conditions. These conclusions are also in line with the actual clinical treatments and observations. On the other hand, the OSI metrics (cf. Figure 8B) suggest a proximal occlusion for case 2, and a distal occlusion for case4, which are contradictory to the metrics of blood stasis as well as clinical observations.

## Discussion

Improving blood-stealing phenomena is the primary goal of CAF treatment, and it is effective in maintaining long-term outcomes by reducing the risk of post-treated fistula thrombosis. This study shows both distal and proximal aneurysm occlusions can reduce the blood flow of fistula, and the percentage of ascending aorta blood flow all increased to close normal value (96%), which corresponds well with our prior study (Cao et al., 2020). On the other hand, the indication of anticoagulation is the most critical issue for occluded CAF patients. According to the American Heart Association (AHA) guidelines, postoperative anticoagulation is required if the maximum diameter of the fistula is greater than 8 mm (Sengupta et al., 2012). As shown in Table 1, the average fistula diameters of four patients were all above this value, requiring a long-term anticoagulation for all patients. However, the fistula is a complex structure, the maximum diameter is not sufficient to determine the risk of thrombosis, and there are currently no suitable criteria for the selection of the occluded position (proximal or distal to the aneurysm). Therefore, it is crucial to find a method to evaluate the degree of thrombosis in the fistula. In our previous study (Cao et al., 2020), the proportion of high OSI areas (>0.3) were used as a metric for risk of thrombosis. Nonetheless, this study shows that area of high OSI other than percentage is a more proper metric for thrombosis, and more accurate for inter-patient comparison.

This study also employed two metrics of blood stasis, i.e. OBVF and OBV, to evaluate thrombosis potential in the fistula. The predicted location of blood residue was roughly consistent with clinical follow-ups for post-treated patients, and also with the high OSI regions. Moreover, the differences in the absolute values (area of high OSI, OBV) between the aneurysm-reserved and aneurysm-removed models were more pronounced than the percentage values (proportion of areas of high OSI, OBVF). The reason for this is that the percentage values are normalized using the surface area or volume of fistula, which also decreased if the aneurysms are removed. Therefore, the absolute values may reflect the degree of thrombosis risk more accurately than the percentage values.

The treatments type decided upon the OBVs is roughly in line with the actual clinical treatment. Nonetheless, this study also shows that OBV is superior to the area of high OSI in determining treatment type (proximal or distal occlusion). This problem can be understood from the nature of the OSI, which is an indicator of oscillatory flows, and believed to play roles in the vessel remodeling and plaque development in arteries (Katritsis et al., 2012). On the other hand, the primary cause of CAF thrombosis is blood stagnation and stasis in the regions proximal to dead ends, which is a “volumetric” process. Therefore, OSI as a metric defined at the wall to account for plaque growth, might not be appropriate to reflect the “volumetric” thrombosis in the fistula, and may misjudge the risk of thrombosis. In contrast, the OBV, a volumetric parameter reflecting the degree of blood stasis, is a more appropriate metric for thrombosis in the fistula. In the future, clinicians may choose an appropriate occluded position according to the predicted fistula OBVs of different treatments. If the OBVs between the two surgical treatments is significantly different, proximal occlusion should be directly selected. If not, morphological parameters need to be involved to opt for the most appropriate treatment based on the relative size of the aneurysm.

### Limitation

This study also has some limitations. First, thrombosis is a very complex process, this study only considered one element in Virchow’s triad, i.e. blood stasis. Since the flow is largely in a stagnant state, endothelial injury due to high WSS is unlikely to happen here. The last factor, i.e. hypercoagulability might influence the thrombosis, and should be considered in the future. Second, this study did not consider the non-Newtonian properties of blood, because it has been reported to hardly affect the hemodynamic parameters of the coronary arteries (Kabinejadian and Ghista, 2012; Vimmr et al., 2013; Frolov et al., 2016). Third, there are two types of multiphase flows, namely, disperse flows and separated flows (Brennen, 2005). The formation of thrombosis is more of disperse flows, while the interaction of the new and old blood over time is more of separated flows. The model of blood stasis employed in study do not model the formation of thrombosis directly, but model the blood stasis which we believe is the primary cause of the thrombosis in the occluded coronary arterial fistulas. Nonetheless, the VOF method used in this study to model blood stasis can handle both separate and dispersed flows. Fourth, since this study is purely retrospective, the four patients investigated in this study only had CTA images shortly after operations. Clinical follow-ups at more time points can better follow the process of thrombosis, which will provide better reference for thrombosis prediction. Moreover, as a retrospective study, patient-specific waveforms were not available. In the future, patient-specific flow rates should be measured and a sensitivity analysis for boundary conditions should be conducted in the follow-up study. Finally, the number of patients investigated in this study is limited, more patients will be needed in future research to make the results statistically significant. In the future, more patients and more CTA images at various time points will be collected to provide better reference for thrombosis prediction. *In vitro* experiments such as microfluidics and animal experiments are also planned to improve our findings.

## Conclusion

In conclusion, the blood stasis model is more intuitive and accurate to simulate the thrombosis risk of postoperative fistulas than the OSI and TAWSS. Together with morphological parameters, the OBV could guide clinicians to formulate more appropriate surgical plans, and provide an efficient non-invasive method for evaluating the risk of thrombosis in the post-treated fistula, which is of great significance for the preoperative evaluation and treatment prognosis of CAF patients.

## Data Availability

The raw data supporting the conclusion of this article will be made available by the authors, without undue reservation.
